# Perceived Electronic Health Record Usability as a Predictor of Task Load and Burnout Among US Physicians: Mediation Analysis

**DOI:** 10.2196/23382

**Published:** 2020-12-22

**Authors:** Edward R Melnick, Elizabeth Harry, Christine A Sinsky, Liselotte N Dyrbye, Hanhan Wang, Mickey Todd Trockel, Colin P West, Tait Shanafelt

**Affiliations:** 1 Department of Emergency Medicine Yale University School of Medicine New Haven, CT United States; 2 University of Colorado School of Medicine Aurora, CO United States; 3 Professional Satisfaction and Practice Sustainability American Medical Association Chicago, IL United States; 4 Department of Medicine Physician Well-Being Program Mayo Clinic Rochester, MN United States; 5 Department of Medicine Stanford School of Medicine Palo Alto, CA United States; 6 Department of Psychiatry and Behavioral Sciences Stanford University School of Medicine Palo Alto, CA United States; 7 Department of Internal Medicine Mayo Clinic Rochester, MN United States; 8 Department of Health Sciences Research Mayo Clinic Rochester, MN United States

**Keywords:** electronic health record, EHR, usability, cognitive load, System Usability Scale, physician task load, NASA Task Load Index, physician, burnout, stress

## Abstract

**Background:**

Electronic health record (EHR) usability and physician task load both contribute to physician professional burnout. The association between perceived EHR usability and workload has not previously been studied at a national level. Better understanding these interactions could give further information as to the drivers of extraneous task load.

**Objective:**

This study aimed to determine the relationship between physician-perceived EHR usability and workload by specialty and evaluate for associations with professional burnout.

**Methods:**

A secondary analysis of a cross-sectional survey of US physicians from all specialties was conducted from October 2017 to March 2018. Among the 1250 physicians invited to respond to the subsurvey analyzed here, 848 (67.8%) completed it. EHR usability was assessed with the System Usability Scale (SUS; range: 0-100). Provider task load (PTL) was assessed using the mental demand, physical demand, temporal demand, and effort required subscales of the National Aeronautics and Space Administration Task Load Index (range: 0-400). Burnout was measured using the Maslach Burnout Inventory.

**Results:**

The mean scores were 46.1 (SD 22.1) for SUS and 262.5 (SD 71.7) for PTL. On multivariable analysis adjusting for age, gender, relationship status, medical specialty, practice setting, hours worked per week, and number of nights on call per week, physician-rated EHR usability was associated with PTL, with each 1-point increase in SUS score (indicating more favorable) associated with a 0.57-point decrease in PTL score (*P*<.001). On mediation analysis, higher SUS score was associated with lower PTL score, which was associated with lower odds of burnout.

**Conclusions:**

A strong association was observed between EHR usability and workload among US physicians, with more favorable usability associated with less workload. Both outcomes were associated with the odds of burnout, with task load acting as a mediator between EHR usability and burnout. Improving EHR usability while decreasing task load has the potential to allow practicing physicians more working memory for medical decision making and patient communication.

## Introduction

Occupational burnout in physicians is a complex phenomenon with multiple interdependent drivers related to practice efficiency, organizational culture, and personal resilience [1]. In the domain of practice efficiency, there is growing evidence supporting an association between electronic health record (EHR) usability and physician burnout [2-7]. Usability is the extent to which technology can be used effectively, efficiently, and satisfactorily based on its design and integration into a specific context of use [8]. Although the EHR has been lauded as a solution to improve health care quality and safety, there is increasing evidence that EHR usability can cause harm [9-12]. Current EHR usability challenges have resulted in systems that many find unnecessarily complex and prone to error, thereby increasing physician cognitive load and resulting in technological errors that can sometimes reach the patient [10-13]. Partially due to excessive time spent on EHR activities, EHR usability is a specific source of physician dissatisfaction and stress [7,14-16]. The topic has received increasing attention since a 2017 systematic review demonstrated a paucity of published studies and standardized reporting on EHR usability evaluation [17-20]. A recently published cross-sectional national survey of physicians from all specialty disciplines identified a strong association between higher physician-perceived EHR usability (assessed by the industry standard, the System Usability Scale [SUS]) and lower odds of physician burnout [7].

In addition to usability, the clerical burden associated with documentation, order entry, inbox management, and other EHR administrative tasks (eg, prior authorizations, documentation of care consistent with quality measures) that are not necessarily intrinsic to the practice of medicine also contributes to excessive time spent on the EHR, as well as unnecessary cognitive burden [16,21,22]. Administrative tasks such as these are independent of EHR design—indeed, the EHR may offer advantages by providing a systematic structure and record to such tasks [21]. According to cognitive load theory, tasks like these can overwhelm limited working memory, the process our minds use to input and respond to all information [23]. Cognitive load refers to the amount of working memory used and comprises three components: intrinsic (complexity of the task itself), extraneous (how the task is presented), and germane (the workload of learning the task or content) load [24,25]. When users are overloaded, data is “shed” or lost, which puts users at risk of committing an error [26]. Using the mental demand, physical demand, temporal demand, and effort required subscales of the National Aeronautics and Space Administration Task Load Index (NASA-TLX), a national survey of 4622 physicians conducted by our team identified a strong association between provider task load (PTL) and burnout but did not assess EHR usability [27,28]. Two studies found enhanced EHR usability is associated with reduced physician cognitive load in simulated EHR environments [29,30].

**Figure 1:**
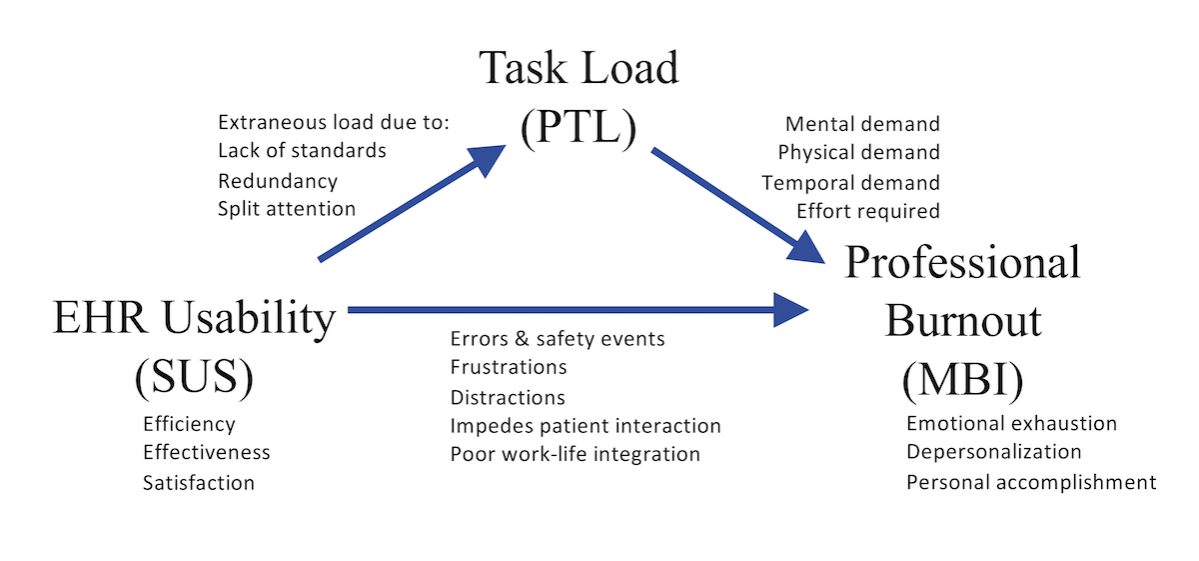
Conceptual framework with provider task load (PTL) as a mediator between electronic health record (EHR) usability and professional burnout. MBI: Maslach Burnout Inventory; SUS: System Usability Scale.

We propose a conceptual framework in which task load acts as a mediator between EHR usability and professional burnout (Figure 1). For example, a poor EHR interface with patient lab values may present redundant data on the screen, creating unnecessary extraneous load that increases the mental demand of a physician’s task. Ongoing exposure to this interface could contribute to burnout for some physicians. Independent of this task, the poor usability could impede patient communication, thereby diminishing the physician’s sense of purpose in their work, thus leading to burnout [10,31].

To our knowledge, the association between perceived EHR usability and workload has not previously been studied at a national level. We conducted a cross-sectional analysis of a national survey to determine the association between physician-perceived EHR usability (using the SUS) and the workload of a clinical workday stratified by specialty and practice setting (using the mental demand, physical demand, temporal demand, and effort required subscales of the NASA-TLX, henceforth referred to as PTL). We hypothesized that greater EHR usability scores (as measured by the SUS) would correlate with lower PTL scores. Also, given that both the SUS and PTL have been shown to relate to physician burnout, we hypothesized that they would both remain associated with burnout after adjusting for personal and professional characteristics. Better understanding these interactions could give further information as to the drivers of extraneous task load.

## Methods

### Study Design, Setting, Participants, and Data Collection

**Figure 2:**
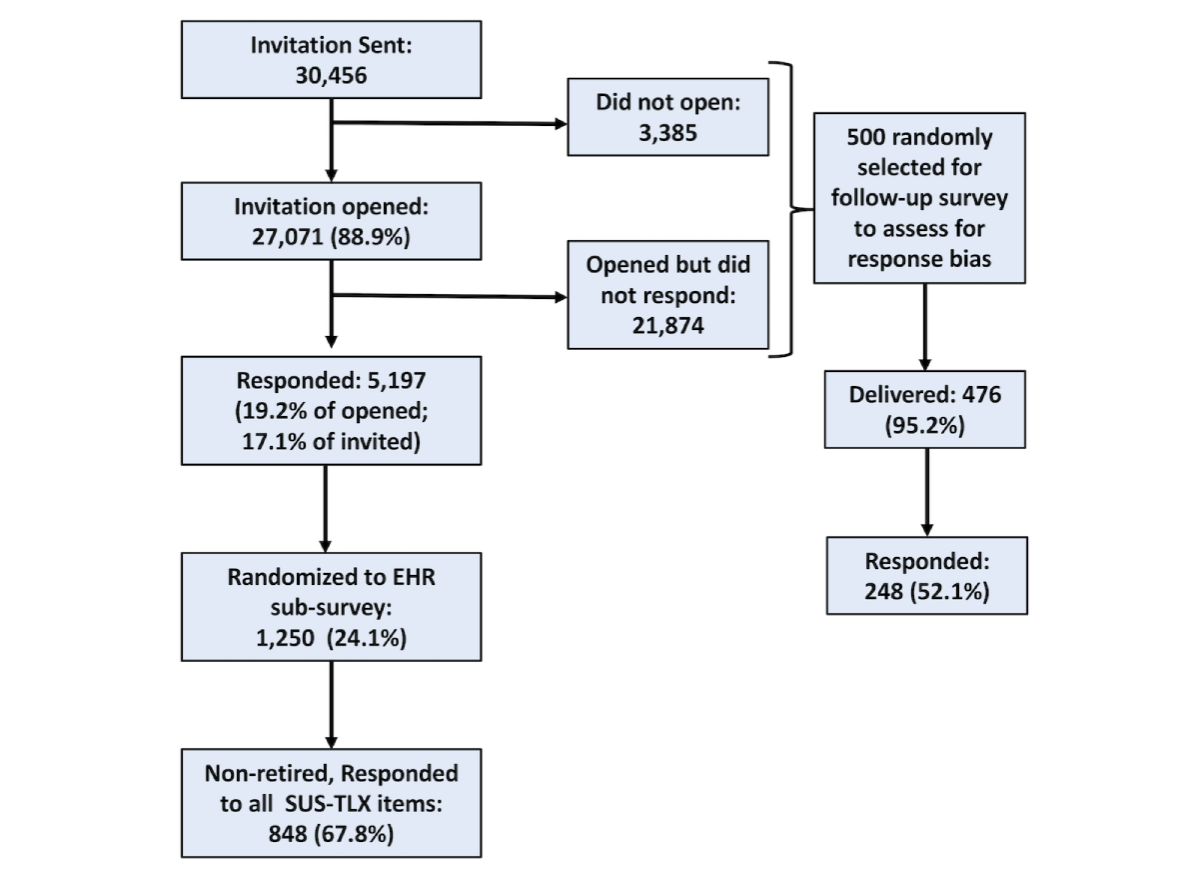
CONSORT (Consolidated Standards of Reporting Trials) diagram of subject enrollment, inclusion criteria, and exclusion criteria. EHR: electronic health record; SUS-TLX: System Usability Scale-Task Load Index.

A secondary analysis of a cross-sectional wellness study of US physicians from all subspecialties was performed. The original survey collected data between October 12, 2017, and March 15, 2018. The sample was assembled using the American Medical Association Physician Masterfile, a nearly complete record of all US physicians, independent of American Medical Association membership. Participation involved voluntary completion of an anonymous electronic survey. Full details of the sampling strategy, recruitment, data collection, and assessment for response bias have previously been reported [32]. Briefly, 30,456 physicians from the Masterfile were invited to participate (Figure 2). Of these, 5197 (17.1%) physicians participated in the study [32]. A random group of 1250 of the participants was invited to complete a subsurvey evaluating their EHR’s usability. Responders who were retired from clinical practice were excluded from the analysis. To evaluate for response bias, an intensive follow-up survey was conducted in a sample of nonresponders. There were 248 (52.1% of 476 invited) participants in the follow-up survey. The Stanford University and Mayo Clinic institutional review boards reviewed and approved the study protocol.

### Study Measures

Study measures included items pertaining to participants’ demographic characteristics (age, gender, relationship status), medical specialty, hours worked per week, number of nights on call per week, practice setting, symptoms of burnout, and perception of their EHR’s usability and daily clinical workload.

### EHR Usability

Physician-perceived EHR usability was measured using the SUS, an industry standard for a quick, reliable measurement of technology usability [33-35]. The SUS includes 10 items, each on a 5-point Likert scale from Strongly Disagree to Strongly Agree that is scored from 0 to 4, with each even-numbered question reverse coded. The items are summed and then multiplied by 2.5 to normalize scores from 0 to 100, with higher scores indicating higher usability. Consistent with convention [34,36], the language of the SUS was modified such that references to “the system” being evaluated were changed to “my EHR.”

### Provider Task Load

Provider task load was measured using 4 items (the mental demand, physical demand, temporal demand, and effort required subscales) from the NASA-TLX [28]. The rationale for exclusion of the frustration and performance NASA-TLX items from our measurement of PTL is that on principal component analysis with oblimin rotation and Kaiser normalization to determine underlying patterns between the 6 items of the NASA-TLX and the emotional exhaustion and depersonalization scales of the Maslach Burnout Inventory, frustration and performance clustered as one component along with emotional exhaustion and depersonalization scores, suggesting that these domains of the NASA-TLX are measures of work-related distress and would be expected to be collinear with burnout measures. Inclusion of the mental demand, physical demand, temporal demand, and effort required NASA-TLX items is consistent with previous reports assessing workload of physicians and nurses [27,37]. Respondents were prompted to “reflect on a day [they] performed clinical work during the last 1-2 weeks that is representative of a typical current clinical work day” and rate their perception of each subscale demand type on a scale of 0 to 100 (with 100 being the highest level of demand) [38,39]. The 4 scores were summed for a composite score ranging from 0 to 400 [40].

### Burnout

Burnout was measured using the validated criterion standard, the Maslach Burnout Inventory [41-44]. Respondents with a high score on the emotional exhaustion (≥27) or depersonalization subscale (≥10) were considered to have at least 1 symptom of burnout [41,45-47].

### Statistical Analysis

Standard descriptive statistics were used to characterize the physician sample that responded to the EHR usability subsurvey and the PTL items of the survey. Associations between variables were evaluated using the Wilcoxon rank sum test (continuous variables) or *χ*^2^ test (categorical variables), as appropriate. Univariable linear regression was used to examine the association between EHR usability and PTL. On preliminary analysis controlling for medical specialty, specialties with smaller numbers of participants in the subsurvey had considerable variability. To control for this variation, specialties with fewer than 20 participants were grouped together in a pooled category of “Other” specialties. The Other category included these specialties (number of respondents in parentheses): neurosurgery (9), ophthalmology (8), otolaryngology (10), other (18), physical medicine and rehabilitation (15), preventive medicine & occupational medicine (4), radiation oncology (5), and urology (7). Two multivariable linear regression analyses were performed to investigate whether the relationship between SUS score and burnout is mediated by PTL. Demographic and professional factors included in the multivariable regression analyses were age, gender, relationship status, hours worked per week, medical specialty, nights on call, and practice setting. All tests were 2-sided, with a type I error level of .05. Analyses were completed using R statistical software (R Foundation for Statistical Computing, Vienna, Austria, Version 3.6.0) with the exception of the mediation analyses, which were conducted using the PROCESS version 3 macro for SPSS (IBM Corporation) [48].

## Results

### Participants

There were 5197 responders to the full survey. A randomly selected group of 1250 of these responders received the EHR usability subsurvey. Among these responders, the 848 individuals (67.8%) who responded to all SUS and PTL items were included in the present analysis. The demographic characteristics (age, gender, and medical specialty) of the respondents included in this analysis were generally similar to the full survey respondents and US physicians (Table 1).

**Table 1:** Demographic characteristics of survey responders.

Characteristic		EHR usability subsurvey responders (N=848)
**Gender, n (%)**		
	Male	493 (58.1)
	Female	348 (41.0)
	Other	1 (0.1)
	Missing	6 (0.7)
Age (years), median (IQR)		53.0 (42.0, 61.0)
**Age (years), n (%)**		
	<35	61 (7.2)
	35-44	200 (23.6)
	45-54	183 (21.6)
	55-64	259 (30.5)
	>/=65	123 (14.5)
	Missing	22 (2.6)
**Specialty, n (%)**		
	Anesthesiology	36 (4.2)
	Dermatology	23 (2.7)
	Emergency medicine	54 (6.4)
	Family medicine	55 (6.5)
	Radiology	37 (4.4)
	Neurology	32 (3.8)
	Obstetrics and gynecology	42 (5.0)
	Pathology	25 (2.9)
	Psychiatry	51 (6.0)
	Other	76 (9.0)
	General internal medicine	77 (9.1)
	Internal medicine subspecialty	113 (13.3)
	General pediatrics	46 (5.4)
	Pediatric subspecialty	53 (6.2)
	General surgery	33 (3.9)
	General surgery subspecialty	58 (6.8)
	Orthopedic surgery	35 (4.1)
	Missing	2 (0.2)
Hours worked per week, median (IQR)		50.0 (40.0, 60.0)
**Hours worked per week, n (%)**		
	<40 h	126 (14.9)
	40-49 h	171 (20.2)
	50-59 h	215 (25.4)
	60-69 h	187 (22.1)
	70-79 h	76 (9.0)
	>80 h	70 (8.3)
	Missing	3 (0.4)
Nights on call per week, median (IQR)		1.0 (0.0, 2.0)
**Primary practice setting, n (%)**		
	Private practice	388 (45.8)
	Academic medical center	274 (32.3)
	Veterans hospital	16 (1.9)
	Active military practice	9 (1.1)
	Other	160 (18.9)
	Missing	1 (0.1)
**Relationship status, n (%)**		
	Single	105 (12.4)
	Married	687 (81.0)
	Partnered	39 (4.6)
	Widowed/widower	8 (0.9)
	Missing	9 (1.1)

### SUS and PTL Scores

**Figure 3:**
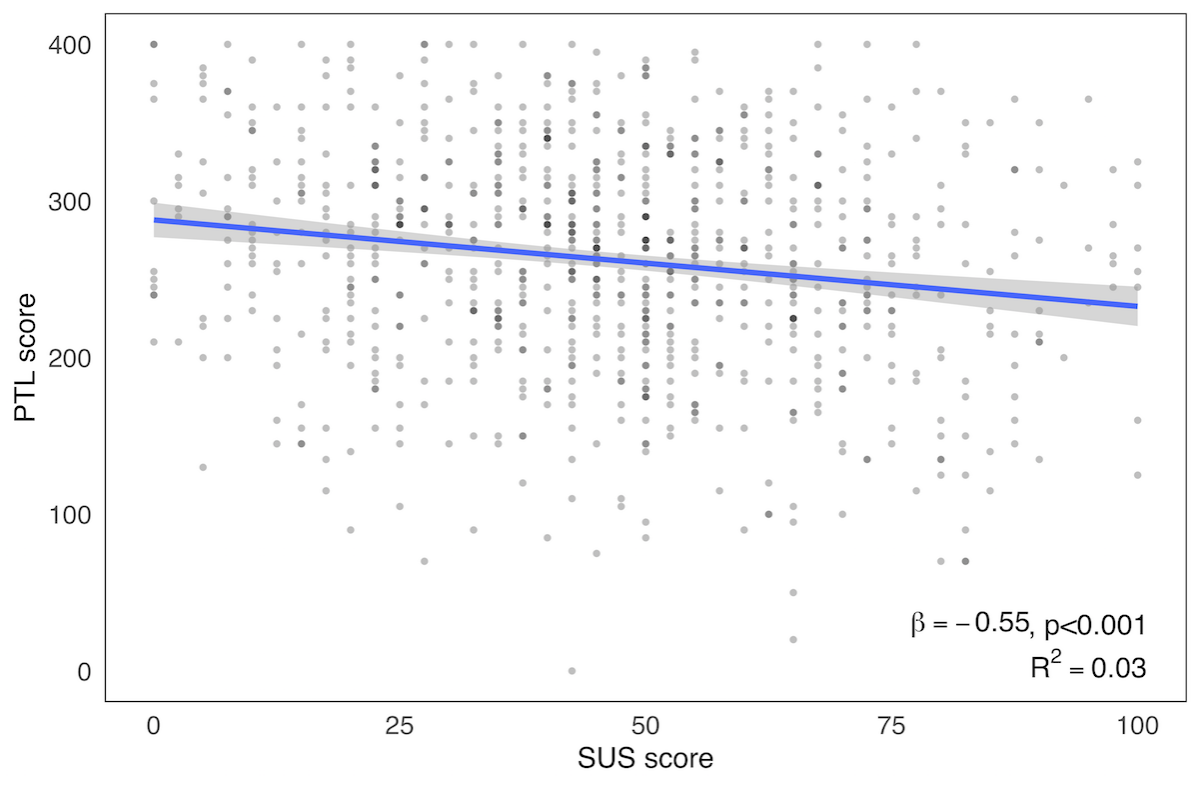
Scatter plot of provider task load (PTL) sum scores (range: 0-400) by System Usability Scale (SUS) scores (range: 0-100) with regression line.

**Figure 4:**
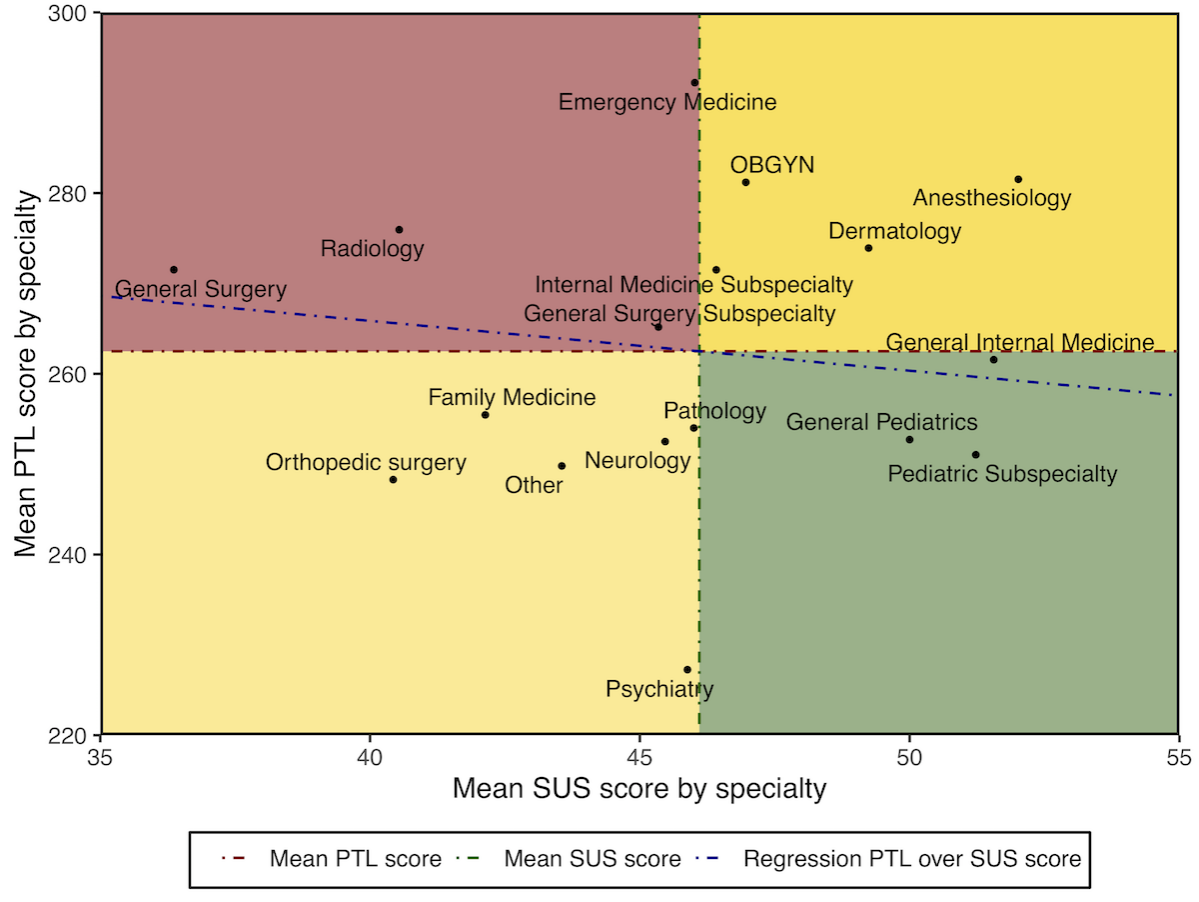
Scatter plot of provider task load (PTL) as assessed by the modified NASA-TLX and electronic health record usability as assessed by the System Usability Scale (SUS) by specialty with regression line. Note that higher SUS indicates more favorable usability, whereas higher PTL indicates increased task load (less favorable).

Physician-perceived EHR usability and PTL scores were similar to those previously reported in the primary analyses of this survey [7,27]. For this analysis, the mean SUS score was 46.1 (SD 22.1; range: 0-100; IQR 30-62.5), and the mean composite PTL score was 262.5 (SD 71.7; range: 0-400; IQR 215-315) with mean subscale scores of 70.9 (SD 20.8) for mental demand, 47.8 (SD 27.3) for physical demand, 68.3 (SD 24.9) for time demand, and 75.6 (SD 21.1) for effort required. On univariate analysis, each 1-point increase in SUS score (indicating greater EHR usability) was associated with a 0.55-point decrease in PTL score (*P*<.001; Figure 3). The relationship between perceived EHR usability and composite PTL scores is shown for the 17 specialty discipline categories in Figure 4.

### Multivariable and Mediation Analyses

On multivariable analysis adjusting for age, gender, relationship status, medical specialty, practice setting, hours worked per week, and number of nights on call per week, EHR SUS scores were associated with PTL. Each 1-point increase in SUS score was associated with a 0.57-point decrease in composite PTL score (*P*<.001; Table 2). Gender; age; practicing emergency medicine, anesthesiology, or psychiatry; hours worked per week; and number of nights on call per week were also associated with composite PTL scores in this model. Being female, practicing emergency medicine or anesthesiology, more hours worked per week, and more nights on call per week were all associated with higher PTL as a measure of workload, whereas being older and practicing psychiatry were both associated with lower workload.

**Table 2:** Predictors of provider task load in multivariable linear regression models among practicing physicians in 2017.

Predictor		Coefficient (SE)	*P* value
SUS^a^ score		−0.57 (0.03)	.001
**Gender (reference: male)**			
	Female	12.59 (1.68)	.03
	Missing/other	19.26 (10.24)	.58
Age, for each year older		−0.45 (0.07)	.048
**Specialty (reference: general internal medicine)**			
	Anesthesiology	30.6 (4.18)	.03
	Dermatology	13.68 (5.03)	.42
	Emergency medicine	47.04 (3.83)	<.001
	Family medicine	−2.87 (3.71)	.82
	Radiology	13.33 (4.25)	.36
	Neurology	−1.18 (4.4)	.94
	Obstetrics and gynecology	9.1 (4)	.5
	Pathology	−4.13 (4.83)	.8
	Psychiatry	−29.11 (3.84)	.03
	Other	−9.66 (3.42)	.4
	Internal medicine subspecialty	8.83 (3.13)	.41
	General pediatrics	−0.76 (3.99)	.96
	Pediatric subspecialty	−14.01 (3.84)	.28
	General surgery	−1.23 (4.4)	.93
	General surgery subspecialty	−6.76 (3.71)	.59
	Orthopedic surgery	−9.47 (4.24)	.51
Hours worked per week, for each additional hour		0.98 (0.05)	<.001
Nights on call per week, for each additional call		3.26 (0.37)	.01
**Primary practice setting (reference: private practice)**			
	Academic medical center	−0.62 (1.77)	.92
	Veterans hospital	2.22 (5.22)	.9
	Active military practice	−31.44 (6.9)	.18
	Other	4.5 (2.07)	.52
**Relationship status (reference: single)**			
	Married	14.12 (2.24)	.06
	Partnered	19.95 (3.93)	.13
	Widowed/widower	46.33 (8.61)	.11

^a^SUS: System Usability Scale.

**Figure 5:**
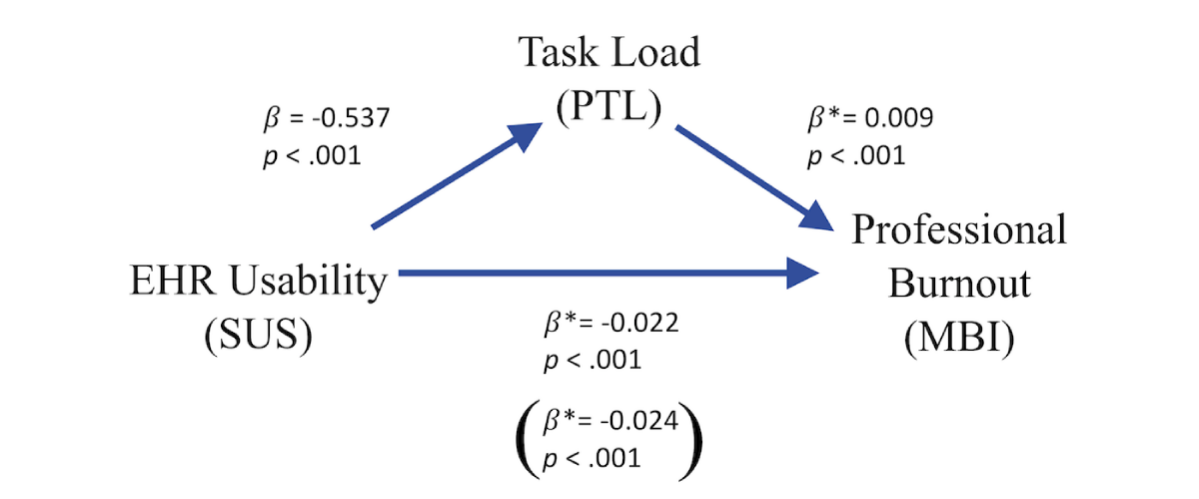
Mediation analysis results showing the quantitative relationships between provider task load (PTL) as a mediator between electronic health record (EHR) usability and professional burnout. The values for EHR usability to professional burnout display the association between the System Usability Scale (SUS) and Maslach Burnout Inventory (MBI) after controlling for PTL and, parenthetically, the association between SUS and MBI when PTL is not statistically controlled for. *log-odds, since this is a logistic regression.

In the first ordinary least squares regression model of the mediation analysis (Figure 5), higher SUS was significantly related to lower PTL scores (β=−.537, 95% CI −0.755 to −0.319; SE 0.111; *P*<.001). In the second logistic regression model, which included SUS and PTL as predictor variables of burnout, both SUS and PTL were significantly associated with burnout (OR 0.978, 95% CI 0.972 to 0.985 and OR 1.009, 95% CI 1.007 to 1.011, respectively). The bootstrap confidence intervals derived from 5000 samples indicated that the indirect effect of PTL on the association between SUS and burnout was significant (effect=−0.005, 95% CI −0.007 to −0.003). From this result, higher SUS was associated with lower PTL, which was associated with lower odds of burnout.

## Discussion

### Principal Results

The findings of this national study indicate that physician-perceived EHR usability scores are strongly associated with physician daily task load. After adjusting for multiple personal and professional characteristics, individual physicians’ composite PTL scores were 0.55 points lower for each 1 point more favorable EHR usability, as measured by the SUS. The mediation analysis supports the proposed conceptual framework in which task load acts as a mediator between EHR usability and professional burnout. Despite the strong statistically significant associations found, the amount of variability in PTL as a measure of workload explained by EHR usability was small (R^2^=0.03). This finding indicates that factors other than EHR usability appear to be the primary drivers of physician workload as assessed by the mental demand, physical demand, temporal demand, and effort required subscales of the NASA-TLX. In the primary analysis of this survey, age, gender, medical specialty, hours worked per week, number of nights on call per week, and practice setting were all associated with PTL as a measure of workload [27]. To put these findings in context, a 10-point lower PTL was associated with 30% lower odds of burnout in the primary analysis [27]. An 18-point higher average SUS score (associated with a 10-point lower PTL in this analysis) would give the EHR a grade of D (instead of F) and be above the score of Microsoft Excel (Microsoft Corporation, 2009) [7].

Exploring this relationship and variation in EHR usability and PTL as a measure of workload by specialty reveals that some specialties with higher task load rated their EHRs more favorably (eg, anesthesiology and dermatology) while other specialties associated with lower task load (eg, orthopedic surgery and family medicine) rated their EHRs less favorably. This finding suggests the relationship between EHR usability and task load may not be due to physicians in specialties with higher task load rating their EHR less favorably. For example, anesthesiologists may benefit from the EHR’s ability to provide certain information rapidly in the operating room, yet still have higher task load due to factors in the anesthesiology clinical context that are not EHR-related. However, only three specialties (emergency medicine, anesthesiology, and psychiatry) remained predictive of task load after controlling for EHR usability, gender, age, hours worked per week, and number of nights on call per week. The analysis is likely underpowered to explain specific differences between individual specialties. On balance, SUS appears to have only a small influence on task load, although the effect of SUS on PTL varies by specialty.

### Comparison With Prior Work

It is also notable that in this analysis, lower EHR usability and higher workload were both associated with the odds of burnout after controlling for multiple personal and professional characteristics, suggesting that these are distinct domains that both represent potential improvement targets to reduce physician burnout. A study of 46 participants’ perception of the usability and task load of three popular websites found no association between usability and task load as assessed by the SUS and NASA-TLX [49]. Important differences between that study and the present study were that SUS scores were only in the high range (compared to the EHR) and that they measured both domains for individual tasks as opposed to in aggregate as we have here. The primary analysis of this national survey of 5197 physicians found that age, gender, relationship status, hours worked per week, and practicing certain medical specialties were all associated with the odds of burnout [32]. In this analysis, gender was not significantly predictive of the odds of burnout. Given this finding and the growing literature on the association of gender on physician burnout [32,50,51], future work should explore the interactions between these variables and further evaluate how EHR usability and task load vary by personal and professional characteristics. For example, a mixed-methods assessment of 25 intensive care physicians running simulated cases found gender-based differences in perceived EHR workload stress, satisfaction, and usability as assessed by the NASA-TLX and SUS [52]. Modest redesign of computerized reminders was associated with improved usability and decreased mental workload for 16 nurses in a simulated environment as assessed by the NASA-TLX [53]. A randomized crossover trial of 7 pediatric surgeons reported improved SUS scores and lower cognitive workload scores when order sets were systematically developed [54]. Future work could explore the relationship between EHR usability, specific task load, and professional burnout across and between different medical specialty disciplines and EHR vendor products as well as further differentiate the contribution of administrative burden to these areas by specialty. Policy makers should also explore the potential savings to the health care system that might be realized by improving EHR usability and how removing required administrative tasks that are not intrinsic to the practice of medicine could decrease task load and burnout.

### Limitations

This study is subject to several limitations. First, although the association between SUS and PTL scores is statistically significant, cross-sectional data cannot prove causation or the potential direction of effect. Second, with all survey research, response bias and representativeness of the study sample are potential limitations. An assessment for response bias was employed in this study, including evaluation of a random sample of nonresponders to the initial survey who participated in an incentivized follow-up survey. Although women were more likely to participate in the EHR usability subsurvey, participants were generally representative of US physicians with respect to age, years in practice, and prevalence of burnout. Third, self-reported data is subject to limitations [55,56]. Physician perceptions of usability and workload may not accurately reflect reality [20]. For example, one dimension of usability is error tolerance, a system’s ability to prevent or recover from an error; due to poor usability, EHR users may not be aware of errors they make in the EHR [11,12]. In addition, individual respondents could have a tendency to provide similar responses to different questions, which could contribute to a common method bias [57]. Fourth, although the SUS and NASA-TLX are considered the industry standards to assess technology usability and task load, they are intended to assess a specific task within a single system, not an entire class of software or an entire day of clinical work. Instead, this analysis provides a global assessment of EHR usability and provider task load and, therefore, cannot account for differences between specific tasks or vendors’ products and may be subject to recall bias. Fifth, physician respondents could conflate EHR usability issues with regulatory and clerical demands that manifest in the EHR but may be unrelated to EHR user interface design [21,22]. If that is the case, the association of clerical burden with task load and burnout could be stronger than that of EHR usability [7,16].

Despite these limitations, as the first national study exploring the relationship between EHR usability and workload in practice across medical specialties and practice settings, this analysis adds an important dimension to existing knowledge about factors associated with PTL and physician burnout. Our findings are consistent with multiple smaller studies of task load in both simulated [29,30] and real world settings [58,59] suggesting that more usable EHR interfaces are associated with lower cognitive load. Technology changes physicians’ information gathering and reasoning strategies, thereby shaping cognitive behavior [60]. Cognitive load theory should be taken into consideration to design EHR interfaces and workflows that meet users’ needs and are free from unnecessary extraneous cognitive load, such that the EHR becomes part of a physician work environment more closely resembling a “manageable cockpit” that is streamlined, ergonomic, and safe [10,61,62].

### Conclusion

As assessed by US physicians using standardized metrics of technology and workload, a strong association was observed between EHR usability and task load, with more favorable usability associated with lower task load. Although EHR usability was significantly related to workload, the effect size was small, indicating that factors other than the EHR appear to be the primary drivers of workload. Both outcomes were also associated with the odds of burnout. Efforts to address physician burnout should attend to both improving EHR usability and addressing other drivers of task load, particularly extraneous load. Improving EHR usability while decreasing extraneous task load has the potential to allow practicing physicians more available working memory for medical decision making and patient communication. Specific areas to target could include consolidating the display of related information, reducing redundancy of information, and increasing standardization [63,64].

## Abbreviations

EHR: electronic health record
NASA-TLX: National Aeronautics and Space Administration Task Load Index
PTL: provider task load
SUS: System Usability Scale
